# The Role of the Oral Microbiome in Circulating Metabolic Biomarkers and the Influence of Air Pollution

**DOI:** 10.1111/odi.70197

**Published:** 2026-01-06

**Authors:** Jiajun Luo, Yuqing Wu, Habibul Ahsan, Christopher O. Olopade, Jayant M. Pinto, Briseis Aschebrook‐Kilfoy

**Affiliations:** ^1^ Department of Public Health and Medicinal Administration, Faculty of Health Sciences University of Macau Macao SAR China; ^2^ Institute for Population and Precision Health, The University of Chicago Biological Science Division Chicago Illinois USA; ^3^ Department of Family Medicine The University of Chicago Biological Science Division Chicago Illinois USA; ^4^ Department of Public Health Sciences The University of Chicago Biological Science Division Chicago Illinois USA; ^5^ Department of Surgery The University of Chicago Biological Science Division Chicago Illinois USA

**Keywords:** air pollution, insulin, oral microbiome

## Abstract

**Aims:**

The oral microbiome is at the frontline for environmental exposure and plays an important role in human metabolism. This study explores the relationship between PM_2.5_ exposure, the oral microbiome, and metabolic biomarkers including ghrelin, resistin, and insulin.

**Methods:**

Data from 473 adult participants (97.7% Black; median age: 53.6) were analyzed. PM_2.5_ exposure was retrospectively assigned based on residential addresses, metabolic biomarkers were measured from blood samples, and oral microbiome profiles were obtained from saliva samples. Multivariate linear regression, weighted quantile sum regression, and high‐dimensional mediation analysis were employed to estimate microbiome‐biomarker associations, the association of the oral microbiome mixture, and mediation effects for PM_2.5_ exposure.

**Results:**

A total of 20 oral microbiome taxa were significantly associated with at least one biomarker, with genus *Atopobium* linked to all three. Insulin demonstrated the strongest sensitivity to the oral microbiome influence. Genera in phyla Actinomycetota and Bacillota played key roles in the relationship between the oral microbiome and metabolic biomarkers. Mediation analysis revealed that the oral microbiome mediated 16.5% and 11.1% of PM_2.5_'s associations with resistin and insulin, respectively.

**Conclusion:**

This study suggests potential mechanisms regarding how the oral microbiome influences metabolic biomarkers and mediates the metabolic effects of PM_2.5_ exposure.

## 1 Background

The relationship between the oral microbiome and metabolism is an emerging area of research that links oral health to systemic conditions. Alterations in the oral microbiome are linked to oral diseases (e.g., periodontitis, caries), which may further lead to metabolic disorders, such as diabetes and obesity, through diverse pathways (e.g., inflammatory and metabolic changes) (Deo and Deshmukh 2019; Sampaio‐Maia et al. 2016; Verma et al. 2018). Therefore, understanding the role of the oral microbiome in disease development could help identify early biomarkers for systemic metabolic disorders as well as preventive interventions for major, burdensome chronic diseases.

Insulin, resistin, and ghrelin are critical indicators of metabolic health, particularly in relation to diabetes (Meier and Gressner 2004). Understanding the relationship between the oral microbiome with these specific biomarkers could provide novel insights into the pathophysiology of metabolic disease and offer potential avenues for therapeutic intervention. Uncovering the complex relationship between the oral microbiome and metabolic biomarkers is also critical for health disparities research because it can uncover how social, environmental, and systemic inequities impact health outcomes across different populations (Renson et al. 2019). We know that limited access to dental care, poor nutrition, and environmental stressors contribute to oral microbiome dysbiosis; these disparities in oral health could be directly linked to systemic conditions that disproportionately affect at‐risk populations (Bastos et al. 2018; Knorst et al. 2021; Thomson 2012). Investigating the oral microbiome and its connection to metabolic biomarkers in diverse populations specifically may help identify population‐specific biomarkers of risk for chronic diseases, develop culturally tailored interventions and oral health policies, and promote precision medicine that considers oral health, systemic health, and social factors.

Standing at the frontline against air pollutants, the oral microbiome is persistently influenced by these exposures. For instance, exposure to fine particulate matter (PM_2.5_) has been shown to impair oral immune defenses and induce microbial dysbiosis, characterized by reduced diversity and altered composition (Lin et al. 2022). Similarly, ozone exposure is associated with decreased microbial diversity and a lower abundance of specific taxa (e.g., Proteobacteria and Firmicutes), which may indicate systemic inflammation (Hu et al. 2020). Beyond the local effects at oral cavities, air pollution has established links to metabolic disorders (An et al. 2018; Rajagopalan and Brook 2012; Zang et al. 2021), suggesting that the oral microbiome can act as a mediator between air pollution exposure and systemic metabolic disorders (Adler et al. 2021; Vieceli et al. 2023; Zhang et al. 2023). As such, understanding the role of environmental stressors like air pollution in the oral microbiome–metabolic biomarker relationship could guide policies and/or interventions. Duration‐sensitive associations between PM_2.5_ exposure and metabolic biomarkers have been established in prior studies, which were hypothesized as mechanistic pathways for the effects of PM_2.5_ exposure (Luo et al. 2024). The extent to which the oral microbiome can influence the adverse impacts of PM_2.5_ exposure remains a crucial area of investigation.

Within this context, this study aims to explore the dual role of the oral microbiome in influencing circulating metabolic biomarkers and modulating the impact of air pollution on these biomarkers. This study is based on data from the Chicago Multiethnic Prevention and Surveillance Study (COMPASS). The goal was to advance our understanding of the complex interactions between the host, microbiome, and environment. We hypothesized that chronic exposure to air pollutants disproportionately impacts the oral microbiome and metabolic health, worsening systemic diseases like diabetes and cardiovascular disease.

## 2 Research Design and Methods

### 2.1 Study Population

COMPASS is an ongoing longitudinal cohort study focused on the South Side of Chicago. During participants' first visit to the research clinic, participants complete the survey questionnaire and provide access to their health records. Anthropometry and blood pressure are then measured and biospecimens, including blood, urine, and saliva, are also collected. Covariates including age, gender (male, female), smoking status (current, former, never), body mass index (BMI; < 18.5, 18.5–24.9, ≥ 25), diabetes status (yes, no), and race (Black/African Americans, White, other) were retrieved from physical examination records and questionnaires. A detailed description of the study design can be found elsewhere (Aschebrook‐Kilfoy et al. 2020).

Among 7409 eligible COMPASS, 650 were randomly selected. Metabolic biomarkers, including insulin, resistin, and ghrelin, were measured in blood samples (Luo et al. 2024) and the oral microbiome was profiled in saliva samples. The participants included in this study were enrolled in COMPASS between 2015 and 2019, thus were not impacted by COVID‐19. All the participants lived at their current addresses for more than 3 years.

### 2.2 PM_2.5_ Exposure Assessment

Ambient PM_2.5_ exposure data was obtained from the Atmospheric Composition Analysis Group at Washington University at St. Louis that estimated the surface PM_2.5_ levels for 1998–2021 through a combination of a satellite‐based model and the GEOS‐Chem chemical transport model and subsequently calibrated to global and North America ground‐based observations (Van Donkelaar et al. 2021). The most updated high resolution (0.01° × 0.01°) datasets for surface PM_2.5_ levels in North America can be obtained online: https://sites.wustl.edu/acag/datasets/surface‐pm2‐5/.

We assigned PM_2.5_ exposure to each participant 1 year prior to the date when they were enrolled according to their residential addresses. This 1‐year exposure duration was chosen based on prior work where we observed that circulating metabolic biomarkers were most sensitive to this time frame (Luo et al. 2024). In the analysis, the PM_2.5_ exposure level was *z*‐score normalized.

### 2.3 Circulating Metabolic Biomarker Measurement

Plasma levels of ghrelin, resistin, and insulin were analyzed by multiplex assays using Human Custom ProcartaPlex 12‐Plex immunoassay (Life Technologies Corp, CA, USA). Briefly, plasma samples were vortexed and spun down. Twenty‐five microliters of the plasma and standards were added to each well of a 96‐well plate. Then assay buffer and multiplexed bead solution were added as per manufacturer's protocol. After a 2‐h incubation at room temperature, the detection antibody mixtures were added to the plate followed by addition of Streptavidin–Phycoerythrin solution. After incubation and washing, the plate was analyzed on a Luminex 200 instrument (Luminex Corporation, Austin, Texas). The median fluorescent intensity (MFI) was used to determine the concentration of the analytes against the standard curve. A four‐parameter logistic (4PL) curve was used to generate the standard curve. All the measures were in pg/mL. In the analysis, all circulating metabolic biomarkers were log transformed, followed by a *z*‐score normalization (Luo et al. 2024).

### 2.4 DNA Extraction and 16s rRNA Amplicon Sequencing of Saliva Samples

Saliva samples (4 mL) were collected from the participants by spitting directly into sterile tubes and immediately frozen at −80°C when the participants visited the research clinic. DNA extraction methods were based on the NIH Manual of Procedures for Human Microbiome Project (Dashper et al. 2019). DNA in saliva was extracted using PowerLyzer PowerSoil DNA Isolation Kits (Qiagen), following the manufacturer's alternate “Vacuum Protocol” with Precellys bead homogenizer (Bertin Technologies). Extracted DNA was quantified using Qubit double stranded DNA High Sensitivity assay kit (Life Technologies), per supplier instructions (Thermo Fisher Scientific, 2015).

Dada2 (v1.18.0) was used as the default pipeline for processing MiSeq 16S rRNA reads with minor modifications in R (v4.0.3). Specifically, reads were first trimmed at 190 bp for both forward and reverse reads to remove low‐quality nucleotides. Chimeras were detected and removed using the default consensus method in the dada2 pipeline. Then, ASVs with lengths between 320 and 365 bp were kept and deemed as high quality ASVs. Taxonomy of the resultant ASVs was assigned to the genus level using the RDP classifier (v2.13) and trainset 18 (release 11.5) with a minimum bootstrap confidence score of 80. Relative ASV abundance was determined by dividing the count associated with that taxon by the total number of filtered reads. Samples with depths below 1000 reads were removed due to insufficient sequencing depths (Kuczynski et al. 2010). To ensure robust taxonomic classification, we analyzed the data at the genus level, as the 16S rRNA V4 region does not typically provide sufficient resolution for reliable species‐level assignment.

### 2.5 Statistical Analysis

First, we described the structure and diversity of the oral microbiome through α and β diversities. The α diversity was calculated as the Shannon–Weiner Index, which reflects both microbial richness and evenness (Sandoval et al. 2019). We used multivariate linear regression to evaluate the association of the Shannon–Weiner Index with PM_2.5_ exposure and each of the three circulating metabolic biomarkers (ghrelin, resistin, insulin). All models were adjusted for age, gender, smoking status, BMI, diabetes status, and household income. The β diversity was measured as Bray–Curtis dissimilarity (Bray and Curtis 1957). We used non‐metric multidimensional scaling (NMDS), which is commonly regarded as the most robust unconstrained ordination method, to analyze and visualize the overall microbiome compositional difference between participants based on the Bray–Curtis dissimilarity (Minchin 1987). We applied the Fast Adonis algorithm to estimate variability in the β diversity explained by PM_2.5_ exposure with adjustment for all covariates.

Second, we used a novel methodology, Analysis of Compositions of Microbiomes with Bias Correction 2 (ANCOM‐BC2) (Lin and Peddada 2024), which can handle the zero‐inflated and compositional microbiome data, to examine the association of each oral microbiome taxon abundance with PM_2.5_ exposure and the three circulating metabolic biomarkers, respectively. ANCOM‐BC2 can perform differential abundance analysis for continuous exposure variables while correcting both the sample‐specific (sampling fraction) as well as taxon‐specific (sequencing efficiency) biases and adjusting for other covariates. ANCOM‐BC2 addresses zero counts by conducting a sensitivity analysis to assess the impact of different pseudo‐counts on zero counts for each taxon. We followed the ANCOM‐BC2 guideline by excluding taxa with a prevalence less than 10% in the analysis. The Benjamini–Hochberg procedure was used to adjust *p* values (Benjamini and Hochberg 1995). A study‐wide false discovery rate (FDR) < 0.05 was considered significant. The same set of covariates as in the first step was adjusted in this step.

Third, we used weighted quantile sum (WQS) regression, a novel mixture analysis method, to integrate the diverse microbiome taxa and evaluate the association of their overall mixture on circulating metabolic biomarkers (Renzetti et al. 2023). This approach allows us to simultaneously consider the association between the outcome and single explanatory variable as well as the correlation between explanatory variables. The WQS approach estimates the mixture association and 95% confidence interval (CI) in either positive or negative direction. The direction for each metabolic biomarker would be determined by results from our first analysis. This method also generates a weight for each taxon that represents the contribution of each taxon to the overall mixture association. All these weights sum up to 100%, and a higher weight indicates a larger contribution. The decile of the microbiome relative abundance was used and thus the estimated mixture association should be interpreted as the impact of a one‐decile increase in overall oral microbiome abundance. In the WQS, we used 60% as training dataset and 40% as validation dataset and conducted cross‐validation. Only taxa that demonstrated significance in the second analysis were included in the mixture analysis. The same set of covariates as in the first step was adjusted in this step.

Fourth, to investigate the potential mediating role of multiple oral microbiome taxa in the relationship between PM_2.5_ exposure and circulating metabolic biomarkers, we conducted high‐dimensional mediation analysis with latent variables (Derkach et al. 2019). This approach extends standard causal mediation analysis by constructing latent variables to represent the overall mediating effect of high‐dimensional mediators (i.e., multiple oral microbiome taxa in this study). The total effect of the PM_2.5_ exposure was decomposed into two components: the natural direct effect (NDE), representing the effect not mediated by the microbiome, and the natural indirect effect (NIE), representing the effect mediated through the microbiome. Additionally, this approach allows for assessment of interactions between the exposure and mediators. Only taxa that demonstrated significance for circulating metabolic biomarkers in the second analysis were included in the mediation analysis. The same set of covariates as in the first step was adjusted in this step.

Missing values were observed in some covariates: household income (17.3% missing), BMI (4.0% missing), and diabetes status (0.6% missing). We addressed these using multiple imputation with the random forest algorithm, which effectively models complex interactions to generate plausible values (Shah et al. 2014). We imputed five complete datasets using all covariates and repeated all analyses in the five datasets. We pooled estimates and corresponding standard errors from these datasets according to Rubin's rule that accounts for the uncertainty of the imputation (Royston 2004). Data supporting this study is available upon reasonable request from the corresponding author. All analyses were conducted using R (version 4.4.0).

We conducted several sensitivity analyses to evaluate the robustness of our results. First, we stratified the study population by smoking status (current vs. never/former) and BMI status (< 25 vs. ≥ 25) to examine the effect modification by these two factors. For each stratum, we repeated the WQS regression analysis to estimate the mixture association of the oral microbiome on each metabolic biomarker. We also tested for interaction by including a product term between the WQS index and the stratification variable in the regression model. Second, we included all taxa in the ANCOM‐BC2 analysis, which allows us to detect rare but potentially functionally important taxa. Third, while the ANCOM‐BC2 framework internally assesses the sensitivity of results to different pseudo‐counts, we performed an additional sensitivity analysis to further evaluate the robustness of our findings by adding a uniform pseudo‐count of 0.5 to all observations prior to analysis. Fourth, we used the 3‐year average PM_2.5_ exposure level to test the robustness of our findings to the exposure window.

## 3 Results

A total of 473 participants with valid data on the oral microbiome and circulating metabolic biomarkers from COMPASS were analyzed in this study. The distributions of selected sociodemographic and medical characteristics are presented in Table 1. The study population was predominantly non‐Hispanic Blacks (97.7%) from low‐income households and 10% reported having type 2 diabetes. The prevalence of current smoking and being overweight (BMI ≥ 25) was both over 60%. A total of 485 microbial taxa were identified and included in this study. The top five taxa (relative abundance median) were *Streptococcus* (0.216), *Prevotella* (0.088), *Haemophilus* (0.068), *Veillonella* (0.063), and *Fusobacterium* (0.030). The oral microbiome composition for each participant can be found in Figure S1.

**TABLE 1:** Distribution of selected characteristics in the study population.

Selected characteristics	*N* (%) or median (interquartile range)
Age	53.6 (46.6–60.2)
Race	
Black/African American	462 (97.7)
White	4 (0.8)
Other	7 (1.5)
Gender	
Female	315 (66.6)
Male	158 (33.4)
Smoking status	
Current	308 (65.1)
Former	48 (10.2)
Never	117 (24.7)
Body mass index	
< 18.5	14 (3.0)
18.5–25	129 (27.3)
≥ 25	311 (65.7)
Missing	19 (4.0)
Diabetes status	
Yes	47 (10.0)
No	423 (89.4)
Missing	3 (0.6)
Household income	
Under $15,000	287 (60.7)
$15,000–$35,000	76 (16.1)
Above $35,000	28 (5.9)
Missing	82 (17.3)
PM_2.5_ exposure	8.9 (8.4–9.3)
Biomarker (pg/mL)	
Resistin	318.4 (156.6–648.1)
Ghrelin	2827.0 (1762.9–4774.8)
Insulin	607.6 (315.9–1125.7)
Relative abundance of the top five oral microbiome genera	
*Streptococcus*	0.216 (0.141–0.355)
*Prevotella*	0.088 (0.047–0.142)
*Haemophilus*	0.068 (0.013–0.146)
*Lactobacillus*	0.063 (0.040–0.094)
*Fusobacterium*	0.030 (0.015–0.049)

**FIGURE 1:**
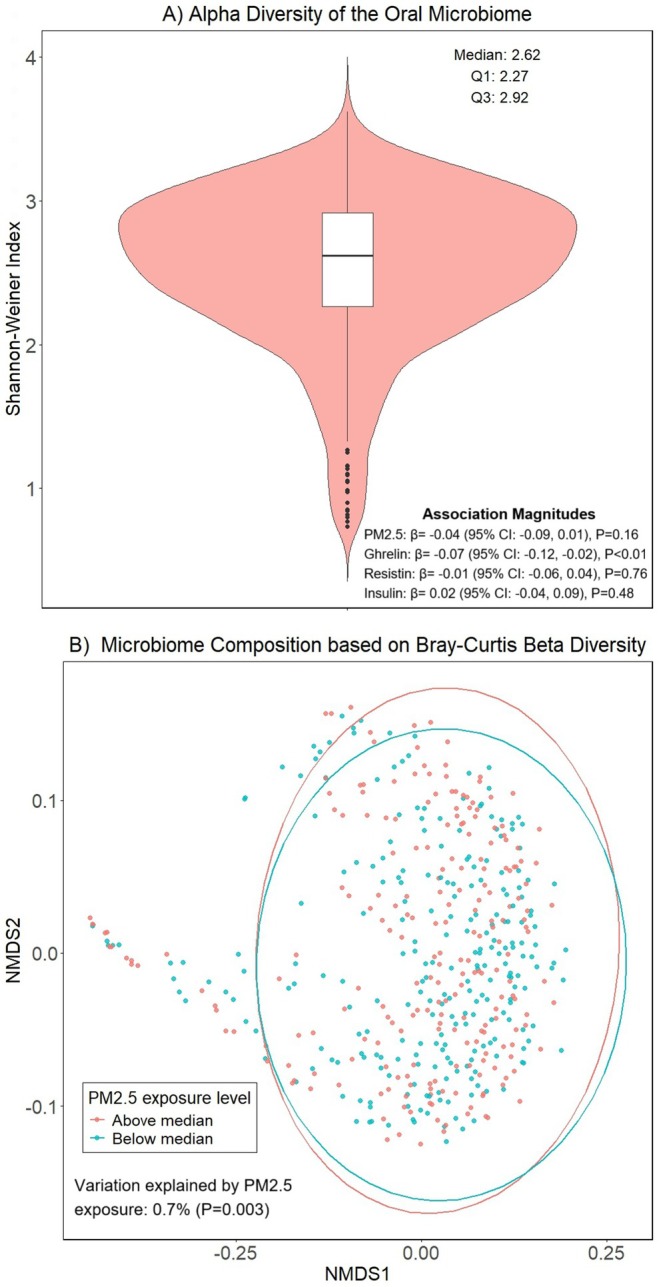
The oral microbiome diversities in the study population. (A) The violin plot of the distribution of oral microbiome alpha diversity. (B) The non‐metric multidimensional scaling (NMDS) plot that represents the oral microbiome composition of each participant based on Bray‐Curtis beta diversity grouped by PM_2.5_ exposure median.

The median oral microbiome α diversity measured as Shannon–Weiner index was 2.62 (interquartile range: 2.27–2.92). When adjusted for covariates, the α diversity was not significantly associated with PM_2.5_ exposure (*β* [95% CI]: −0.04 [−0.09, 0.01]; *p* value: 0.16) (Figure 1). Similarly, no significant association was observed between the α diversity and the circulating levels of resistin (*p* value: 0.76) or insulin (*p* value: 0.47); however, ghrelin was significantly associated with the α diversity (*β* [95% CI]: −0.07 [−0.12, −0.02]; *p* value < 0.01). A small but significant percentage (0.7%, *p* value: 0.003) of the overall variability in β diversity was explained by PM_2.5_ exposure in this study population. The full model that adjusted for all covariates explained a modest percentage (6.0%) of the overall variability in β diversity (Figure 1).

**FIGURE 2:**
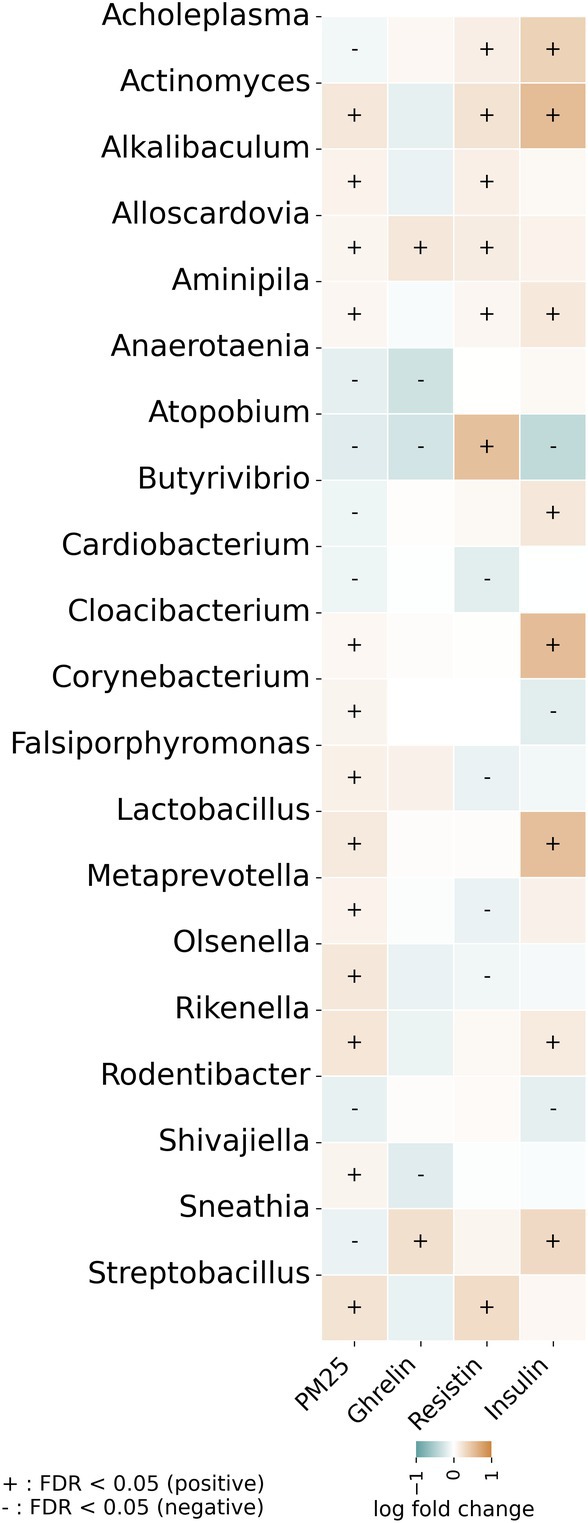
Associations between oral microbiome genera, PM_2.5_ exposure, and metabolic biomarkers. The heatmap displays the log_2_ fold change in genus abundance associated with a one‐unit increase in PM_2.5_ (*z*‐score) or biomarker level (*z*‐score), as determined by ANCOM‐BC2. Only genera with at least one significant association (false discovery rate [FDR] < 0.05) are shown. Red indicates a positive association (increase), and blue indicates a negative association (decrease). The intensity of the color corresponds to the magnitude of the log_2_ fold change. A plus sign (+) denotes a significant positive association, and a minus sign (−) denotes a significant negative association (FDR < 0.05).

The associations between significant oral microbiome taxa, PM_2.5_ exposure, and the three metabolic biomarkers are visualized in Figure 2 (association magnitudes are detailed in Table S1). This heatmap shows the direction and strength (log_2_ fold change) of these associations, with highlights of statistically significant results (FDR < 0.05). As detailed below, several key patterns emerge. A total of 20 taxa showed significant associations (FDR < 0.05) with at least one circulating metabolic biomarker. Ghrelin was significantly associated with five taxa, with *Anaerotaenia* and *Atopobium* exhibiting the strongest associations. Resistin was significantly positively associated with 11 taxa, with *Atopobium* and *Actinomyces*, plus *Streptobacillus*, showing the strongest associations. Insulin was significantly associated with 12 taxa. Among these taxa, nine showed positive associations, with *Actinomyces*, *Cloacibacterium*, and *Lactobacillus* showing the strongest associations, while three taxa exhibited inverse associations. Notably, only one taxon, *Atopobium*, was significantly associated with all three biomarkers, while six taxa were associated with two biomarkers. All these 20 taxa were significantly associated with PM_2.5_ exposure.

When considering the oral microbiome as a mixture so that we could estimate mixture association, we observed significant findings for all three metabolic biomarkers (Table 2). For ghrelin, the mixture association was *β* = −1.39 (95% CI: −1.77, −1.01), with *Atopobium* contributing 69.1% to the mixture association. In contrast, the mixture associations for resistin and insulin were positive, as *Atopobium* contributed most to the mixture impact as well with a weight of 65.0% and 42.9%, respectively. For resistin, the mixture association was *β* = 1.24 (95% CI: 0.83, 1.65). Besides *Atopobium*, another genus, *Streptobacillus* also contributed substantially to the mixture association on resistin, with a weight of 16.8%. Insulin had an association of *β* = 0.88 (95% CI: 0.63, 1.13). Two other genera, *Acholeplasma* (23.3%) and *Lactobacillus* (11.2%), also contributed substantially.

**TABLE 2:** Mixture association of the oral microbiome on the circulating metabolic biomarkers and the contribution of individual microbiome taxa.

	Ghrelin	Resistin	Insulin
Mixture effect (95% CI)^a^	−1.39 (−1.77, −1.01)	1.24 (0.83, 1.65)	0.88 (0.63, 1.13)
Contribution weight of microbiome taxa^b^ in the mixture effect			
*Acholeplasma*	0	0.6%	23.3%
*Actinomyces*	0	0.3%	9.3%
*Alkalibaculum*	0	3.9%	0
*Alloscardovia*	2.0%	5.8%	0
*Aminipila*	0	< 0.1%	1.5%
*Anaerotaenia*	4.7%	0	0
*Atopobium*	69.1%	65.0%	42.9%
*Butyrivibrio*	0	0	0.4%
*Cardiobacterium*	0	< 0.1%	0
*Cloacibacterium*	0	0	4.8%
*Corynebacterium*	0	0	0.4%
*Falsiporphyromonas*	0	< 0.1%	0
*Lactobacillus*	0	0	11.2%
*Metaprevotella*	0	3.8%	0
*Olsenella*	0	3.9%	0
*Rikenella*	0	0	3.4%
*Rodentibacter*	0	0	< 0.1%
*Shivajiella*	22.7%	0	0
*Sneathia*	1.6%	0	3.9%
*Streptobacillus*	0	16.8%	0

Abbreviation: CI, confidence interval.

^a^ The mixture effect is interpreted as one decile increase in the microbiome mixture.

^b^ This table only presents taxa with a contribution weight > 1%.

**FIGURE 3:**
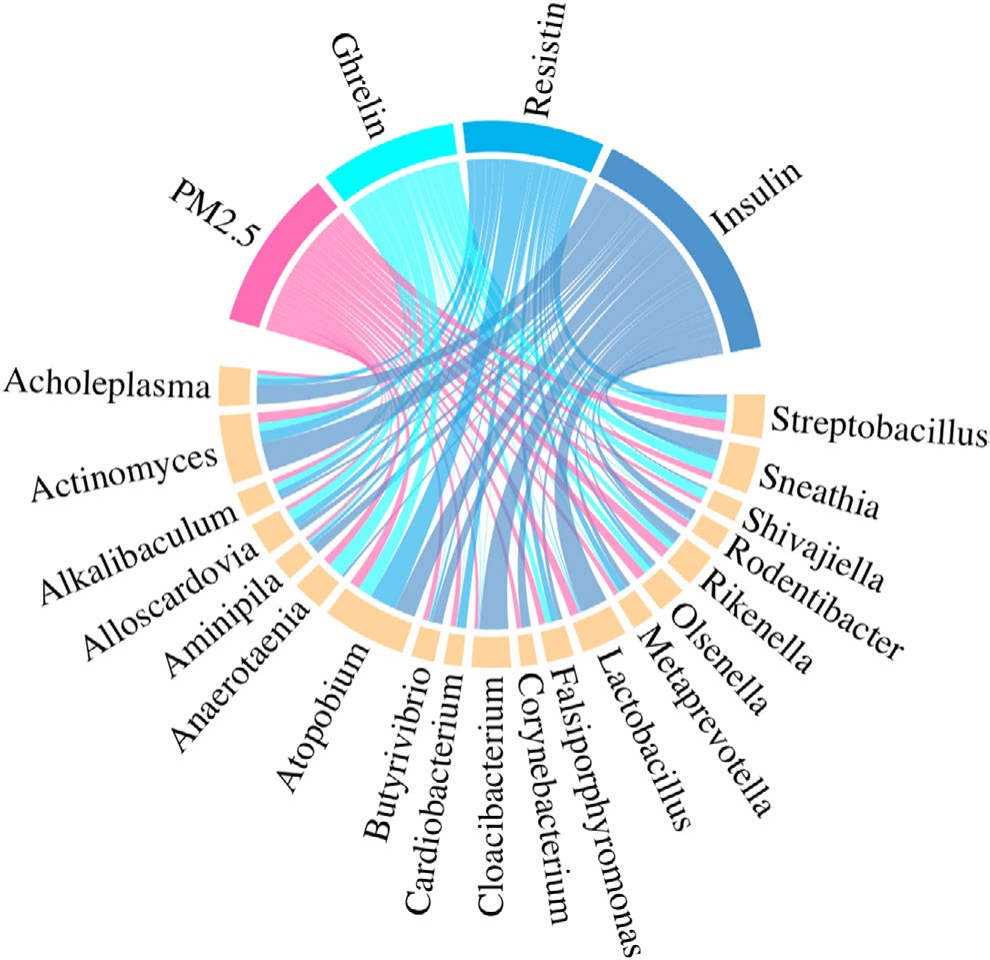
Mediation pathways linking PM_2.5_ exposure to metabolic biomarkers through the oral microbiome. The circos plot visualizes the significant mediation pathways identified by the high‐dimensional mediation analysis. The outer ring segments represent the variables: PM_2.5_ exposure (blue), significant microbiome genera (green), and metabolic biomarkers (orange). Ribbons connecting the segments represent significant associations. The width of each ribbon is proportional to the absolute value of the effect size (e.g., the coefficient estimate for the PM_2.5_‐genus association or the mediated effect for the genus‐biomarker pathway).

The overall mediation model, illustrating the interconnected pathways between PM_2.5_, the mediating microbiome taxa, and the metabolic biomarkers, is summarized in Figure 3. In this circos plot, the width of the ribbons connecting the variables corresponds to the magnitude of the association or mediation effect, providing a visual summary of the three‐way relationships among PM_2.5_ exposure, microbiome taxa, and circulating biomarkers identified in this study. We observed significant mediation effects of the oral microbiome in the associations of PM_2.5_ exposure with resistin and insulin, but not ghrelin (Figure 3; Table 3). PM_2.5_ exposure was significantly associated with ghrelin and insulin, with a borderline significance for resistin (Table 3). The NIE mediated by the oral microbiome was significant for resistin and insulin. For ghrelin, the NIE of PM_2.5_ exposure was non‐significant with *β* = 0.01 (95% CI: −0.01, 0.04). For resistin, the total effect was borderline significant (*β* = 0.14, 95% CI: −0.03, 0.30), but the NIE was significant (*β* = 0.02, 95% CI: 0, 0.06), explaining 16.5% (*p* value: 0.03) of the total effect. For insulin, the total effect was *β* = 0.21 (95% CI: 0.04, 0.42), with an NIE of *β* = 0.02 (95% CI: 0, 0.03), which accounted for 11.1% (*p* value: 0.04) of the total effect. No significant interaction between the PM_2.5_ exposure and the oral microbiome was detected.

**TABLE 3:** The mediation analysis results for the three circulating biomarkers.

Metabolic biomarkers	PM_2.5_ exposure		Oral microbiome	Proportion mediated	
	Total effect	Natural direct effect	Natural indirect effect	Proportion (%)	*p*
Ghrelin	−0.42 (−0.57, −0.25)	−0.43 (−0.59, −0.26)	0.01 (−0.01, 0.04)	−2.6	0.28
Resistin	0.14 (−0.03, 0.30)	0.12 (0.01, 0.21)	0.02 (0, 0.06)	16.5	0.03
Insulin	0.21 (0.04, 0.42)	0.19 (0.02, 0.38)	0.02 (0, 0.03)	11.1	0.04

Our sensitivity analyses confirmed the robustness of the primary findings. The associations between the oral microbiome mixture and metabolic biomarkers were consistent in populations stratified by smoking status and BMI (Table S2). Although the estimates suggested stronger associations among current smokers and individuals with a BMI ≥ 25, the interaction terms were not statistically significant (all *p* for interaction > 0.10). Furthermore, the results were robust to variations in analytical methodology. When we included taxa with a prevalence below 10% in the ANCOM‐BC2 analysis, 142 taxa were significantly associated with at least one biomarker, with 15 taxa (including *Atopobium*) associated with all three (Table S3). Notably, *Moraxella*, which had a prevalence below 10% and was excluded from the main analysis, exhibited the strongest associations across all biomarkers. Similarly, when adding a uniform pseudo‐count of 0.5 to all taxa in the ANCOM‐BC2 analysis, we identified 215 significant taxa, with 117 associated with all three biomarkers; among these, *Veillonella* showed the strongest associations (Table S4). Lastly, the main findings were not sensitive to the exposure window, as the 20 significant taxa identified in the main analysis remained significantly associated when using a 3‐year average PM_2.5_ exposure level (Table S5).

## 4 Discussion

This study supports a critical role of the oral microbiome in human metabolism. Additionally, we found that the oral microbiome mediated a significant portion of the association between PM_2.5_ exposure and circulating metabolic biomarkers, highlighting the importance of considering microbial‐mediated mechanisms in understanding the impacts of environmental exposures on metabolic health. To our best knowledge, this is the largest study linking the oral microbiome with human metabolic conditions, especially using circulating biomarkers. Overall, findings from this study revealed complex relationships between environmental exposures, the oral microbiome, and metabolic health. These insights could lead to ways of mitigating the adverse health impacts of air pollution.

*Atopobium* shows consistent associations with PM_2.5_ exposure and all three circulating metabolic biomarkers in this study. Meanwhile, it also contributes most to the mixture impact of the overall oral microbiome on metabolic biomarkers, suggesting the crucial role of *Atopobium* in the oral microbiome in shaping human metabolic health. These findings were supported by limited studies in the literature. *Atopobium*, a genus of commensal bacteria within the phylum Actinomycetota found in the human microbiome of different sites, has been linked to several aspects of metabolic health. In the gut, *Atopobium* strains can directly influence host metabolism by modulating the activity of PPARγ, a nuclear receptor involved in regulating inflammation and energy balance; this effect is mediated through phosphorylation pathways and the production of bioactive metabolites, suggesting a role in maintaining metabolic homeostasis and potentially impacting conditions like obesity or diabetes (Nepelska et al. 2017). In the oral cavity, higher levels of *Atopobium* have been associated with hypertension, as studies show that its abundance in saliva can serve as a predictor of high blood pressure, possibly through effects on sulfur metabolism and the renin–angiotensin system (Murugesan and Al Khodor 2023). Additionally, *Atopobium* has been found in increased abundance in the early stages of colorectal cancer, particularly in individuals with multiple polypoid adenomas and intramucosal carcinomas, indicating a possible link between *Atopobium*, metabolic byproducts, and cancer risk (Vitali et al. 2015). While *Atopobium* is part of the skin, mucosal, oral, and gut microbiota, current research does not specifically document changes in *Atopobium* abundance or activity in response to air pollution. This study is the first one suggesting the complex relationship between air pollution exposure, *Atopobium* in the oral microbiome, and human metabolic health.

We also observed that the phylum Bacillota (e.g., *Lactobacillus*) substantially influenced insulin and mediated the relationship between PM_2.5_ exposure and metabolic biomarkers, which is supported by evidence in the literature. Research shows that various *Lactobacillus* species can help regulate glucose and lipid metabolism, reduce inflammation, and improve gut barrier function, which collectively contribute to the prevention and management of metabolic disorders such as obesity, type 2 diabetes, and metabolic syndrome (Rastogi and Singh 2022; Wang et al. 2021; Yan et al. 2019; Zheng et al. 2021). *Lactobacillus* also influences cholesterol metabolism by regulating genes involved in cholesterol synthesis and absorption, potentially lowering cardiovascular risk (Cao et al. 2021). Emerging research indicates that air pollution, particularly PM_2.5_ exposure, can influence *Lactobacillus* populations and their health‐related effects in humans and animal models. Exposure to PM2.5 has been associated with shifts in microbial communities, including a reduction in *Lactobacillus* dominance (Oyebode et al. 2023). Animal studies show that supplementation with specific *Lactobacillus* strains, such as *L. acidophilus* and *L. casei* , can mitigate the harmful effects of air pollution by reducing inflammation and restoring beneficial bacteria, including *Lactobacillus* itself (Jin et al. 2020; Jung et al. 2022; Yang et al. 2022).

This study highlights the mediating role of the oral microbiome in the relationship between PM_2.5_ exposure and circulating metabolic biomarkers. Previous studies have established that increased PM_2.5_ exposure increases the risk for metabolic disorders (An et al. 2018; Rajagopalan and Brook 2012; Zang et al. 2021) and proposed several potential mechanisms, including increased oxidative stress and adipose tissue inflammation, hepatic lipid accumulation, and decreased glucose utilization in skeletal muscle (An et al. 2018; Rajagopalan and Brook 2012; Zang et al. 2021). Our study extends this understanding by demonstrating, for the first time, that PM_2.5_ influences circulating metabolic biomarkers by altering the abundance of specific oral bacteria, including genera within the phyla Actinomycetota and Bacillota. These findings are consistent with prior evidence showing that Actinobacteria and Bacillota in the oral and respiratory microbiome communities are more prevalent among individuals living in highly polluted areas (Li et al. 2019; Navarro et al. 2024). Notably, our study estimated that over 10% of the impact of PM_2.5_ exposure on resistin and insulin was mediated by the oral microbiome, emphasizing the oral microbiome as a key mechanistic pathway for PM_2.5_ induced metabolic health effects. This novel insight underscores the potential of targeting the oral microbiome to mitigate the adverse health impacts of air pollution and warrants more investigations in this area.

This study primarily focused on prevalent genera; however, our sensitivity analyses that relaxed the prevalence threshold revealed intriguing findings. Specifically, we identified two taxa, *Moraxella* and *Veillonella*, which demonstrated notably strong associations with all three metabolic biomarkers. *Moraxella* is often considered a pathobiont of the respiratory tract (Goldstein et al. 2009). Its presence in the oral cavity may signal a broader disruption of mucosal immunity or reflect inflammatory pathways shared between respiratory and metabolic health (Goldstein et al. 2009). *Veillonella* is known for its role in lactate acid metabolism, with some species linked to systemic inflammatory conditions (Zhang and Huang 2023). The absence of these two genera from the main analysis highlights a critical insight: while focusing on common taxa ensures statistical robustness, rare or low‐prevalence members of the oral microbiome may exert critical influences on host metabolism as well. This suggests that the metabolic influence of the oral microbiome is not limited to a few taxa but may involve a broader, more complex community, including low‐abundance species with potentially significant pathogenic or protective roles.

Our mediation analysis did not detect a statistically significant interaction between the exposure (i.e., PM_2.5_ exposure) and the mediator (i.e., the oral microbiome). Tests for exposure‐mediator interaction typically require a larger sample size than tests for the main mediation effects, particularly in high‐dimensional settings with multiple mediators. Using a recently developed tool specifically for mediation analysis (Qin 2024), our study was underpowered to detect a moderate exposure–mediator interaction. Therefore, we cannot rule out the possibility that the mediating effect of the oral microbiome varies at different levels of air pollution exposure. The significant mediation effects we observed should thus be interpreted as an average effect across the PM_2.5_ exposure range in our cohort. Future studies with larger sample sizes are necessary to investigate potential effect modification by air pollution, which could reveal the microbiome's differentiating role in distinct exposure levels, thereby refining our understanding of this complex pathway.

There are several limitations to consider in this study. First, the generalizability of our findings must be considered in the context of the study population. Our cohort was predominantly non‐Hispanic Black and low‐income, reflecting the community residents of the South Side of Chicago. This is a major strength for health disparities research, as this population is often underrepresented in environmental health studies, yet bears a disproportionate burden of disease. However, it also suggests that the specific estimates of the associations we observed may not be directly generalizable to other racial/ethnic or socioeconomic groups where social, cultural, and genetic factors may differently shape the oral microbiome and metabolic responses. Moreover, the PM_2.5_ exposure levels in our study were relatively low (8–12 μg/m^3^) compared to concentrations found in highly polluted areas such as China or India, where levels can regularly exceed 50 μg/m^3^. Therefore, the magnitude of the associations and the mediation proportions we report may not be applicable to populations experiencing extreme air pollution. These limitations underscore the importance of replicating our findings in more diverse cohorts and across a broader range of environmental exposures to investigate the comprehensive role of oral microbiome in disease development. Second, our analysis was conducted at the genus level due to the limitations of 16S rRNA sequencing for reliable species‐level classification. While this approach identified significant associations with genera like *Atopobium* and *Lactobacillus*, it is possible that more precise biological mechanisms are driven by specific species or strains within these genera, which can have divergent functional roles. Future research utilizing whole‐genome shotgun metagenomic sequencing would be valuable to investigate these relationships at a higher taxonomic and functional resolution. Third, though using circulating metabolic biomarkers was an advantage of this study, the changes in these biomarkers were not linked to specific clinical diagnoses. How these changes may eventually lead to clinical diagnosis and whether any intervention on these biomarkers would benefit the population should be investigated in future studies. Fourth, we lacked data on critical potential confounders, including oral health behaviors (e.g., oral hygiene practices, frequency of dental visits), dietary intake, and medication use that affects the microbiome or metabolism (e.g., antibiotics, metformin). Although we adjusted for socioeconomic proxies like income and smoking, which are correlated with these factors, the possibility of residual confounding cannot be ruled out. Last, this study is cross‐sectional in nature, even though we were able to assign the air pollution level retrospectively. Therefore, caution is needed when we establish a relationship between PM_2.5_ exposure and one‐time biomarker measures. However, we believe the risk of reverse causation is minimal in this context (i.e., the oral microbiome modulates the metabolic activities, not vice versa). The mediation analysis results could still provide insights into the complex relationship between air pollution, the oral microbiome, and human metabolic health.

Our study provides evidence of the oral microbiome's role in mediating the metabolic effects of PM_2.5_ exposure. These findings offer insights for future research. Most importantly, longitudinal studies are needed to track individuals over time, establishing the temporal relationship between PM_2.5_ exposure, dynamic changes in the oral microbiome, and the incidence of metabolic disorders. Additionally, interventional studies are warranted to test whether modifying the oral microbiome, through approaches such as probiotic supplementation, dietary interventions, or enhanced oral hygiene, can effectively mitigate the adverse metabolic impacts of air pollution exposure. Finally, it is critical to replicate these findings in more diverse populations, including groups with different racial/ethnic backgrounds, socioeconomic statuses, and those experiencing a broader spectrum of PM_2.5_ exposure, such as residents of highly polluted regions in Asia and Europe. Confirming these pathways across diverse contexts will be essential for developing targeted public health strategies to improve our understanding of the role of the oral microbiome and reduce the global burden of pollution‐related metabolic disease.

In conclusion, we observed that multiple oral microbiome taxa were associated with circulating metabolic biomarkers (including ghrelin, resistin, and insulin), underscoring the critical relationship between the oral microbiome and human metabolic disorders like obesity and diabetes. We further observed that the oral microbiome mediated a large portion of the association of PM_2.5_ exposure on these circulating metabolic biomarkers, providing novel insights about the mechanisms for the PM_2.5_ exposure.

## Author Contributions

**Jiajun Luo:** conceptualization, methodology, writing – original draft, writing – review and editing, formal analysis, data curation. **Yuqing Wu:** formal analysis, software, visualization, writing – review and editing, data curation. **Habibul Ahsan:** writing – original draft, writing – review and editing, funding acquisition, supervision, resources. **Christopher O. Olopade:** writing – review and editing, resources, supervision, project administration. **Jayant M. Pinto:** writing – review and editing, writing – original draft, supervision, resources, funding acquisition. **Briseis Aschebrook‐Kilfoy:** writing – original draft, funding acquisition, conceptualization, writing – review and editing, resources, supervision, project administration.

## Funding

This research was supported by funding from NIH grants P30ES027792 and 1OT2OD036445.

## Conflicts of Interest

The authors declare no conflicts of interest.

## Supporting information


**Data S1:** odi70197‐sup‐0001‐DataS1.docx.

## Data Availability

The data that support the findings of this study are available on request from the corresponding author. The data are not publicly available due to privacy or ethical restrictions.
