# Supplementary material for: The Role of the Oral Microbiome in Circulating Metabolic Biomarkers and the Influence of Air Pollution
Source: Oral Dis. 2026 Jan 6;32(6):1665–74. doi: 10.1111/odi.70197 (PMC13034858; doi:10.1111/odi.70197)
Supplement: Supplementary file 1 [file ODI-32-1665-s001.docx]

**Title:** The role of oral microbiome in circulating metabolic biomarkers and the influence of air pollution – Supplemental

**Authors and affiliations:** Jiajun Luo, PhD, MPH^1,2,3^, Yuqing Wu, MS^2^, Habibul Ahsan, MD^1,2,3^, Christopher O Olopade, MD^3^, Jayant M. Pinto, MD^4^, Briseis Aschebrook-Kilfoy, PhD, MPH^1,2,3^

^1^ Department of Public Health Sciences, the University of Chicago Biological Science Division, Chicago, USA

^2^ Institute for Population and Precision Health, the University of Chicago Biological Science Division, Chicago, USA

^3^ Department of Family Medicine, the University of Chicago Biological Science Division Chicago, USA

^4^ Department of Surgery, the University of Chicago Biological Science Division Chicago, USA

**Corresponding author**:

Briseis Aschebrook-Kilfoy, PhD, MPH, MPhil

Associate Professor

Institute for Population and Precision Health

The University of Chicago

5841 S. Maryland Ave. | MC 6100, Room TC-620 | Chicago, IL 60637

Office: 773-834-8699

Email: brisa@uchicago.edu

Figure 1. Heatmap for the oral microbiome composition

Table 1. Study-wide significant association between oral microbiome taxa and circulating metabolic biomarkers

| Microbiome taxa | Ghrelin | | Resistin | | Insulin | |
| --- | --- | --- | --- | --- | --- | --- |
|  | Log fold change^a^ | FDR | Log fold change^a^ | FDR | Log fold change^a^ | FDR |
| Acholeplasma | -0.08 | 0.01 | 0.01 | 0.68 | 0.13 | <0.01 |
| Actinomyces | 0.20 | <0.01 | -0.15 | 0.19 | 0.23 | <0.01 |
| Alkalibaculum | 0.10 | 0.01 | -0.13 | 0.14 | 0.13 | 0.01 |
| Alloscardovia | 0.09 | 0.05 | 0.20 | 0.04 | 0.15 | <0.01 |
| Aminipila | 0.07 | 0.02 | -0.04 | 0.85 | 0.07 | 0.04 |
| Anaerotaenia | -0.16 | <0.01 | -0.30 | <0.01 | 0.00 | 0.99 |
| Atopobium | -0.19 | <0.01 | -0.28 | <0.01 | 0.51 | <0.01 |
| Butyrivibrio | -0.11 | <0.01 | 0.01 | 0.52 | 0.06 | 0.30 |
| Cardiobacterium | -0.10 | <0.01 | -0.02 | 0.92 | -0.18 | <0.01 |
| Cloacibacterium | 0.07 | 0.05 | 0.02 | 0.95 | 0.01 | 0.85 |
| Corynebacterium | 0.09 | 0.01 | 0.03 | 0.84 | 0.04 | 0.81 |
| Falsiporphyromonas | 0.12 | <0.01 | 0.12 | 0.26 | -0.13 | <0.01 |
| Lactobacillus | 0.18 | 0.01 | 0.03 | 0.92 | 0.02 | 0.85 |
| Metaprevotella | 0.10 | 0.01 | -0.03 | 0.92 | -0.13 | <0.01 |
| Olsenella | 0.19 | <0.01 | -0.13 | 0.20 | -0.09 | 0.02 |
| Rikenella | 0.21 | <0.01 | -0.12 | 0.21 | 0.06 | 0.08 |
| Rodentibacter | -0.14 | <0.01 | 0.03 | 0.92 | 0.05 | 0.96 |
| Shivajiella | 0.07 | 0.01 | -0.16 | 0.03 | -0.02 | 0.75 |
| Sneathia | -0.13 | <0.01 | 0.24 | 0.05 | 0.08 | 0.07 |
| Streptobacillus | 0.22 | <0.01 | -0.14 | 0.20 | 0.28 | <0.01 |
| FDR, false discovery rate  ^a^ Model adjusted for age, gender (male, female), smoking status (current, former, never), body mass index (BMI; <18.5, 18.5-24.9, ≥25), diabetes status (yes, no), and household income (under $15,000, $15,000-$35,000, above $35,000). | | | | | | |

Table S2. Estimated mixture effect of the oral microbiome on the circulating metabolic biomarkers in populations stratified by smoking status and BMI

| Population characteristics | Mixture effect (95% CI)^a^ | | |
| --- | --- | --- | --- |
|  | Ghrelin | Resistin | Insulin |
| **Smoking** |  |  |  |
| Current (n=308) | -2.01 (-3.70, -0.32) | 1.84 (0.34, 3.34) | 1.23 (0.21, 2.25) |
| Former/never (n=165) | -0.97 (-2.07, 0.13) | 1.02 (-0.12, 2.16) | 0.71 (0.02, 2.69) |
| P for interaction | 0.15 | 0.24 | 0.21 |
|  |  |  |  |
| **BMI** |  |  |  |
| <25 (n=143) | -1.12 (-2.20, -0.04) | 1.02 (0.01, 2.03) | 0.65 (-0.07, 1.37) |
| ≥25 (n=311) | -1.55 (-2.64, -0.46) | 1.42 (0.13, 2.71) | 1.01 (0.21, 1.81) |
| P for interaction | 0.84 | 0.63 | 0.35 |
| CI, confidence interval  ^a^ The mixture effect is interpreted as one decile increase in the microbiome mixture. | | | |

Table S3. Study-wide significant association between oral microbiome taxa and circulating metabolic biomarkers when using all taxa regardless of their prevalence in the study population

| Microbiome taxa | Ghrelin | | Resistin | | Insulin | |
| --- | --- | --- | --- | --- | --- | --- |
|  | Log fold change^a^ | FDR | Log fold change^a^ | FDR | Log fold change^a^ | FDR |
| Acetobacteroides | 0.70 | 0.02 | -0.22 | <0.01 | NS | NS |
| Acholeplasma | -0.08 | 0.01 | NS | NS | 0.13 | <0.01 |
| Acinetobacter | NS | NS | -0.13 | <0.01 | 0.30 | <0.01 |
| Actinobaculum | NS | NS | 0.08 | <0.01 | NS | NS |
| Actinomyces | 0.20 | <0.01 | NS | NS | 0.23 | <0.01 |
| Aerococcus | NS | NS | -0.40 | <0.01 | NS | NS |
| Aggregatibacter | NS | NS | NS | NS | -0.22 | 0.02 |
| Alkalibaculum | 0.10 | 0.01 | NS | NS | 0.13 | 0.01 |
| Allisonella | NS | NS | -0.12 | <0.01 | 0.18 | <0.01 |
| Alloscardovia | 0.09 | 0.05 | 0.20 | 0.04 | 0.15 | <0.01 |
| Alterileibacterium | NS | NS | -0.24 | <0.01 | NS | NS |
| Alysiella | -0.20 | 0.03 | 0.08 | <0.01 | 0.22 | <0.01 |
| Aminipila | 0.07 | 0.02 | NS | NS | 0.07 | 0.04 |
| Anaerobacterium | NS | NS | 0.61 | <0.01 | -2.30 | 0.04 |
| Anaerococcus | 0.21 | 0.02 | NS | NS | -0.49 | <0.01 |
| Anaeroglobus | 0.32 | 0.01 | 0.37 | <0.01 | 0.22 | <0.01 |
| Anaeroglobus | NS | NS | NS | NS | 0.23 | <0.01 |
| Anaeroplasma | NS | NS | 0.09 | <0.01 | 0.26 | <0.01 |
| Anaerorhabdus | -0.21 | 0.02 | NS | NS | 0.26 | <0.01 |
| Anaerotaenia | -0.27 | <0.01 | NS | NS | NS | NS |
| Anaerovibrio | NS | NS | 0.11 | <0.01 | 0.24 | <0.01 |
| Anaerovorax | NS | NS | -0.52 | <0.01 | NS | NS |
| Arachnia | -0.32 | 0.01 | -0.19 | <0.01 | NS | NS |
| Asteroleplasma | 0.53 | <0.01 | -0.51 | <0.01 | -0.90 | <0.01 |
| Atopobium | -0.25 | 0.01 | 0.50 | <0.01 | -0.42 | <0.01 |
| Bacilliculturomica | NS | NS | 0.07 | <0.01 | NS | NS |
| Bacteroides | NS | NS | -0.08 | 0.01 | NS | NS |
| Bariatricus | NS | NS | 0.20 | <0.01 | NS | NS |
| Bifidobacterium | NS | NS | NS | NS | 0.24 | <0.01 |
| Bilophila | 1.00 | <0.01 | -0.87 | <0.01 | NS | NS |
| Brachymonas | NS | NS | 0.23 | <0.01 | NS | NS |
| Brevundimonas | NS | NS | 7.16 | <0.01 | NS | NS |
| Breznakibacter | NS | NS | -0.13 | <0.01 | 0.21 | 0.02 |
| Bulleidia | NS | NS | -0.12 | <0.01 | 0.42 | <0.01 |
| Butyrivibrio | NS | NS | NS | NS | 0.18 | <0.01 |
| Campylobacter | 0.47 | <0.01 | -0.53 | <0.01 | 0.59 | <0.01 |
| Candidatus | NS | NS | -0.10 | <0.01 | -0.12 | 0.02 |
| Cardiobacterium | NS | NS | -0.19 | <0.01 | NS | NS |
| Catabacter | NS | NS | NS | NS | 0.17 | 0.02 |
| Caviibacter | NS | NS | 0.22 | <0.01 | 0.14 | 0.01 |
| Chrysiogenes | 0.58 | 0.01 | -0.10 | <0.01 | NS | NS |
| Citroniella | NS | NS | -0.09 | <0.01 | NS | NS |
| Cloacibacterium | NS | NS | NS | NS | 0.51 | <0.01 |
| Clostridium | NS | NS | NS | NS | -0.14 | 0.02 |
| Conchiformibius | 0.23 | 0.02 | NS | NS | -0.16 | <0.01 |
| Coprococcus | NS | NS | -0.15 | <0.01 | -0.36 | <0.01 |
| Corynebacterium | NS | NS | -0.23 | <0.01 | -0.20 | <0.01 |
| Cryptobacterium | NS | NS | -0.06 | <0.01 | -0.23 | <0.01 |
| Cucumibacter | NS | NS | -0.38 | <0.01 | NS | NS |
| Cutibacterium | 0.20 | 0.03 | NS | NS | NS | NS |
| Desulfobulbus | NS | NS | 0.08 | 0.01 | 0.18 | <0.01 |
| Desulfomicrobium | NS | NS | 0.08 | <0.01 | 0.11 | 0.01 |
| Desulfovermiculus | NS | NS | 0.30 | <0.01 | NS | NS |
| Desulfovibrio | NS | NS | -0.05 | <0.01 | 0.18 | <0.01 |
| Dialister | NS | NS | -0.63 | <0.01 | NS | NS |
| Duncaniella | NS | NS | -0.10 | <0.01 | -0.69 | <0.01 |
| Eggerthia | NS | NS | 0.11 | 0.01 | NS | NS |
| Eikenella | NS | NS | NS | NS | -0.10 | 0.01 |
| Enhydrobacter | 0.20 | 0.05 | -0.12 | <0.01 | -0.22 | <0.01 |
| Eremococcus | NS | NS | 0.13 | <0.01 | 0.18 | <0.01 |
| Euryhalocaulis | NS | NS | -0.11 | <0.01 | NS | NS |
| Exilispira | NS | NS | -0.32 | <0.01 | NS | NS |
| Faecalibaculum | NS | NS | -0.07 | <0.01 | 0.24 | <0.01 |
| Falsiporphyromonas | NS | NS | -0.14 | <0.01 | NS | NS |
| Fannyhessea | NS | NS | -0.46 | <0.01 | NS | NS |
| Faucicola | 0.52 | <0.01 | -0.30 | <0.01 | NS | NS |
| Flexilinea | NS | NS | 0.04 | <0.01 | NS | NS |
| Fudania | NS | NS | -0.06 | 0.01 | NS | NS |
| Geothermobacter | NS | NS | -0.31 | <0.01 | 0.90 | 0.01 |
| Gudongella | -0.24 | 0.01 | -0.11 | <0.01 | NS | NS |
| Howardella | NS | NS | -0.12 | <0.01 | NS | NS |
| Hydrogenimonas | NS | NS | NS | NS | 0.33 | <0.01 |
| Ihubacter | NS | NS | -0.11 | <0.01 | NS | NS |
| Intestinibaculum | NS | NS | -0.38 | <0.01 | -0.19 | <0.01 |
| Klebsiella | NS | NS | -2.11 | 0.01 | NS | NS |
| Lacticaseibacillus | NS | NS | 0.18 | 0.01 | NS | NS |
| Lactobacillus | NS | NS | NS | NS | 0.50 | <0.01 |
| Lactococcus | NS | NS | -0.08 | <0.01 | 0.32 | <0.01 |
| Lactonifactor | NS | NS | -0.12 | <0.01 | -0.13 | <0.01 |
| Ligilactobacillus | NS | NS | NS | NS | 0.53 | <0.01 |
| Limosilactobacillus | NS | NS | NS | NS | 0.37 | <0.01 |
| Mageeibacillus | NS | NS | -0.73 | <0.01 | NS | NS |
| Magnetospira | NS | NS | -0.19 | <0.01 | 0.12 | 0.04 |
| Mailhella | NS | NS | 1.24 | 0.02 | NS | NS |
| Mannheimia | NS | NS | -0.15 | <0.01 | -0.17 | <0.01 |
| Marseilla | NS | NS | 0.61 | 0.04 | NS | NS |
| Mediterranea | NS | NS | -0.13 | <0.01 | -0.29 | 0.01 |
| Megasphaera | -0.20 | 0.04 | -0.25 | <0.01 | NS | NS |
| Metaprevotella | NS | NS | -0.14 | <0.01 | NS | NS |
| Microbacter | 0.36 | <0.01 | -0.42 | <0.01 | NS | NS |
| Millionella | -0.21 | 0.03 | NS | NS | 0.12 | 0.01 |
| Mobiluncus | NS | NS | -0.97 | <0.01 | NS | NS |
| Monoglobus | NS | NS | 0.05 | <0.01 | -0.17 | <0.01 |
| Moraxella | -1.12 | <0.01 | -2.41 | <0.01 | -2.71 | <0.01 |
| Moryella | NS | NS | 0.14 | 0.01 | NS | NS |
| Mucinivorans | NS | NS | -0.11 | <0.01 | 0.15 | <0.01 |
| Mycoplasma | NS | NS | 0.08 | <0.01 | NS | NS |
| Mycoplasmopsis | NS | NS | 0.30 | <0.01 | 0.51 | <0.01 |
| Occidentia | -0.27 | 0.04 | -0.41 | <0.01 | 0.61 | <0.01 |
| Odoribacter | NS | NS | 0.12 | <0.01 | 0.20 | <0.01 |
| Olsenella | NS | NS | -0.09 | <0.01 | NS | NS |
| Parabacteroides | NS | NS | -0.09 | 0.01 | NS | NS |
| Parascardovia | NS | NS | 0.07 | <0.01 | -0.51 | <0.01 |
| Parvimonas | -0.25 | 0.01 | NS | NS | NS | NS |
| Pelobium | 0.21 | 0.03 | 0.06 | <0.01 | 0.25 | <0.01 |
| Pelospora | NS | NS | -0.53 | <0.01 | NS | NS |
| Peptoanaerobacter | NS | NS | -0.07 | 0.04 | NS | NS |
| Peptoniphilus | NS | NS | -0.17 | <0.01 | NS | NS |
| Phocaeicola | NS | NS | 0.28 | <0.01 | 0.23 | <0.01 |
| Phocaeicola | NS | NS | -1.22 | 0.02 | NS | NS |
| Propionibacterium | NS | NS | -0.45 | <0.01 | -0.32 | <0.01 |
| Propionibacterium | NS | NS | -0.60 | <0.01 | NS | NS |
| Pseudomonas | NS | NS | -0.76 | <0.01 | -0.53 | <0.01 |
| Pyramidobacter | NS | NS | NS | NS | 0.12 | 0.01 |
| Rhodocytophaga | NS | NS | -0.69 | 0.03 | NS | NS |
| Rikenella | NS | NS | 0.04 | 0.01 | 0.14 | <0.01 |
| Rodentibacter | NS | NS | NS | NS | -0.19 | <0.01 |
| Rothia | NS | NS | -0.22 | <0.01 | NS | NS |
| Ruficoccus | NS | NS | -0.30 | <0.01 | -0.20 | <0.01 |
| Saccharibacteria | 0.37 | <0.01 | NS | NS | -0.29 | <0.01 |
| Schleiferia | NS | NS | 0.11 | <0.01 | NS | NS |
| Schwartzia | 0.97 | <0.01 | -0.38 | <0.01 | 0.32 | <0.01 |
| Selenomonas | NS | NS | 0.19 | <0.01 | 0.17 | 0.01 |
| Simonsiella | NS | NS | 1.58 | 0.01 | NS | NS |
| Sneathia | 0.28 | <0.01 | NS | NS | 0.29 | <0.01 |
| Snodgrassella | NS | NS | -0.05 | <0.01 | -0.29 | <0.01 |
| Sphaerochaeta | NS | NS | -0.08 | <0.01 | -0.17 | <0.01 |
| Spirochaeta | NS | NS | 0.36 | <0.01 | 0.16 | <0.01 |
| SR1 | 0.30 | 0.01 | -0.30 | <0.01 | NS | NS |
| Staphylococcus | 0.31 | <0.01 | -0.15 | <0.01 | -0.21 | <0.01 |
| Stenotrophomonas | NS | NS | -1.07 | <0.01 | NS | NS |
| Streptobacillus | NS | NS | 0.27 | <0.01 | NS | NS |
| Streptophyta | NS | NS | -0.09 | <0.01 | NS | NS |
| Syntrophococcus | 0.33 | <0.01 | -0.19 | <0.01 | 0.30 | <0.01 |
| Tagaea | -0.32 | 0.01 | 0.20 | <0.01 | NS | NS |
| Thioreductor | -0.55 | <0.01 | -0.24 | <0.01 | 0.27 | <0.01 |
| Tissierella | -0.40 | <0.01 | 0.05 | 0.01 | 0.12 | 0.01 |
| Unknown | NS | NS | NS | NS | 0.27 | <0.01 |
| Veillonella | -0.25 | 0.01 | 0.20 | <0.01 | -0.40 | <0.01 |
| Williamwhitmania | 0.22 | 0.01 | NS | NS | NS | NS |
| Wolinella | NS | NS | -0.50 | <0.01 | NS | NS |
| Zea | NS | NS | NS | NS | 0.43 | <0.01 |
| NS, non-significant; FDR, false discovery rate  ^a^ Model adjusted for age, gender (male, female), smoking status (current, former, never), body mass index (BMI; <18.5, 18.5-24.9, ≥25), diabetes status (yes, no), and household income (under $15,000, $15,000-$35,000, above $35,000). | | | | | | |

Table S4. Study-wide significant association between oral microbiome taxa and circulating metabolic biomarkers when adding a uniform pseudo-count of 0.5 to all observations

| Microbiome taxa | Ghrelin | | Resistin | | Insulin | |
| --- | --- | --- | --- | --- | --- | --- |
|  | Log fold change^a^ | FDR | Log fold change^a^ | FDR | Log fold change^a^ | FDR |
| Abiotrophia | -0.16 | 0.02 | NS | NS | NS | NS |
| Acetatifactor | -0.09 | 0.02 | 0.12 | <0.01 | 0.20 | <0.01 |
| Acetobacter | NS | NS | 0.06 | <0.01 | 0.14 | <0.01 |
| Acetobacteroides | 0.25 | <0.01 | -0.73 | <0.01 | -0.34 | <0.01 |
| Acholeplasma | NS | NS | 0.09 | <0.01 | 0.29 | <0.01 |
| Acidaminococcus | -0.18 | <0.01 | 0.11 | <0.01 | 0.13 | <0.01 |
| Acinetobacter | NS | NS | NS | NS | 0.46 | <0.01 |
| Actinobaculum | 0.18 | <0.01 | NS | NS | NS | NS |
| Actinomyces | -0.15 | 0.02 | 0.23 | <0.01 | 0.50 | <0.01 |
| Aestuariispira | -0.09 | <0.01 | 0.11 | <0.01 | 0.18 | <0.01 |
| Aggregatibacter | NS | NS | NS | NS | -0.21 | 0.02 |
| Agrobacterium | 0.19 | <0.01 | -0.23 | <0.01 | -0.09 | <0.01 |
| Agromyces | NS | NS | 0.04 | 0.04 | NS | NS |
| Akkermansia | -0.09 | <0.01 | 0.11 | <0.01 | 0.18 | <0.01 |
| Algoriphagus | -0.09 | <0.01 | 0.11 | <0.01 | 0.20 | <0.01 |
| Alistipes | -0.09 | <0.01 | 0.11 | <0.01 | 0.18 | <0.01 |
| Alkalibaculum | -0.13 | 0.02 | 0.09 | 0.04 | NS | NS |
| Allisonella | NS | NS | -0.28 | <0.01 | NS | NS |
| Alloscardovia | 0.20 | <0.01 | 0.14 | <0.01 | NS | NS |
| Alterileibacterium | -0.26 | <0.01 | 0.13 | <0.01 | NS | NS |
| Aminicella | -0.14 | 0.01 | 0.12 | <0.01 | 0.26 | <0.01 |
| Anaerocella | NS | NS | NS | NS | -0.07 | 0.02 |
| Anaerococcus | -0.12 | <0.01 | 0.11 | <0.01 | 0.22 | <0.01 |
| Anaerococcus | 0.28 | <0.01 | -0.10 | <0.01 | -0.66 | <0.01 |
| Anaeroglobus | -0.23 | <0.01 | 0.23 | <0.01 | 0.51 | <0.01 |
| Anaeroplasma | -0.20 | <0.01 | 0.09 | <0.01 | 0.22 | <0.01 |
| Anaerorhabdus | -0.23 | <0.01 | -0.15 | <0.01 | NS | NS |
| Anaerosinus | 0.21 | <0.01 | -0.43 | <0.01 | -0.08 | 0.01 |
| Anaerosphaera | NS | NS | 0.04 | 0.01 | NS | NS |
| Anaerotaenia | -0.31 | <0.01 | -0.10 | <0.01 | NS | NS |
| Anaerovorax | -0.35 | <0.01 | 0.36 | <0.01 | 0.76 | <0.01 |
| Apibacter | 0.40 | <0.01 | -0.79 | <0.01 | -0.25 | <0.01 |
| Asaia | -0.11 | <0.01 | 0.12 | <0.01 | 0.20 | <0.01 |
| Atopobium | -0.59 | <0.01 | 0.55 | <0.01 | 1.07 | <0.01 |
| Bacteroides | NS | NS | -0.13 | <0.01 | -0.13 | 0.02 |
| Bariatricus | -0.15 | 0.01 | 0.09 | <0.01 | -0.33 | <0.01 |
| Barnesiella | -0.09 | <0.01 | 0.11 | <0.01 | 0.18 | <0.01 |
| Bergeyella | -0.11 | 0.01 | 0.09 | <0.01 | 0.25 | <0.01 |
| Bifidobacterium | NS | NS | NS | NS | 0.24 | <0.01 |
| Blastocatella | -0.09 | <0.01 | 0.11 | <0.01 | 0.21 | <0.01 |
| Blastomonas | -0.09 | <0.01 | 0.11 | <0.01 | 0.20 | <0.01 |
| Bosea | 0.12 | 0.02 | NS | NS | NS | NS |
| Bryocella | -0.10 | 0.03 | 0.10 | <0.01 | 0.14 | <0.01 |
| Buttiauxella | -0.10 | <0.01 | 0.12 | <0.01 | 0.20 | <0.01 |
| Butyrivibrio | NS | NS | NS | NS | 0.18 | <0.01 |
| Campylobacter | NS | NS | NS | NS | -0.18 | 0.01 |
| Campylobacter | -0.25 | <0.01 | 0.25 | <0.01 | 0.62 | <0.01 |
| Cardiobacterium | NS | NS | -0.23 | <0.01 | NS | NS |
| Catenibacterium | -0.09 | <0.01 | 0.12 | <0.01 | 0.23 | <0.01 |
| Catonella | NS | NS | -0.07 | 0.04 | NS | NS |
| Chryseobacterium | -0.09 | <0.01 | 0.11 | <0.01 | 0.21 | <0.01 |
| Chryseobacterium | 0.23 | <0.01 | -0.54 | <0.01 | -0.17 | <0.01 |
| Cloacibacterium | -0.11 | <0.01 | 0.15 | <0.01 | 0.35 | <0.01 |
| Cloacibacterium | NS | NS | -0.14 | <0.01 | NS | NS |
| Clostridium | NS | NS | NS | NS | -0.12 | 0.04 |
| Clostridium | -0.09 | <0.01 | 0.11 | <0.01 | 0.18 | <0.01 |
| Colidextribacter | -0.13 | <0.01 | 0.14 | <0.01 | 0.37 | <0.01 |
| Conservatibacter | NS | NS | 0.12 | <0.01 | NS | NS |
| Coprobacter | -0.09 | <0.01 | 0.11 | <0.01 | 0.18 | <0.01 |
| Corynebacterium | -0.39 | <0.01 | 0.34 | <0.01 | 0.71 | <0.01 |
| Cryptobacterium | -0.13 | <0.01 | 0.14 | <0.01 | 0.26 | <0.01 |
| Cutibacterium | 0.13 | 0.01 | NS | NS | NS | NS |
| Deinococcus | -0.13 | <0.01 | 0.09 | <0.01 | 0.18 | <0.01 |
| Denitrobacterium | -0.09 | <0.01 | 0.11 | <0.01 | 0.20 | <0.01 |
| Desulfobulbus | -0.23 | <0.01 | NS | NS | 0.21 | <0.01 |
| Dialister | -0.31 | <0.01 | 0.29 | <0.01 | 0.59 | <0.01 |
| Dichelobacter | NS | NS | 0.04 | 0.01 | NS | NS |
| Dorea | -0.09 | <0.01 | 0.11 | <0.01 | 0.18 | <0.01 |
| Eggerthia | NS | NS | NS | NS | -0.17 | 0.02 |
| Eikenella | NS | NS | NS | NS | -0.15 | <0.01 |
| Eisenbergiella | -0.09 | <0.01 | 0.11 | <0.01 | 0.18 | <0.01 |
| Empedobacter | -0.09 | <0.01 | 0.11 | <0.01 | 0.18 | <0.01 |
| Enterobacter | -0.09 | <0.01 | 0.11 | <0.01 | 0.18 | <0.01 |
| Enterococcus | NS | NS | 0.12 | <0.01 | NS | NS |
| Entomoplasma | NS | NS | -0.20 | <0.01 | -0.19 | <0.01 |
| Eremococcus | -0.13 | 0.02 | 0.13 | <0.01 | NS | NS |
| Erwinia | NS | NS | 0.05 | <0.01 | NS | NS |
| Escherichia/Shigella | -0.09 | <0.01 | 0.11 | <0.01 | 0.18 | <0.01 |
| Exiguobacterium | -0.13 | <0.01 | 0.14 | <0.01 | 0.14 | <0.01 |
| Ezakiella | 0.27 | <0.01 | -0.52 | <0.01 | -0.31 | <0.01 |
| Faecalibacterium | -0.09 | 0.05 | 0.08 | <0.01 | 0.19 | <0.01 |
| Faecalibaculum | 0.41 | <0.01 | -0.98 | <0.01 | -0.39 | <0.01 |
| Faecalicoccus | -0.09 | <0.01 | 0.11 | <0.01 | 0.18 | <0.01 |
| Falcatimonas | NS | NS | 0.10 | <0.01 | 0.21 | <0.01 |
| Falsiporphyromonas | NS | NS | -0.12 | <0.01 | -0.15 | 0.01 |
| Filobacterium | NS | NS | -0.06 | <0.01 | NS | NS |
| Finegoldia | -0.09 | <0.01 | 0.11 | <0.01 | 0.18 | <0.01 |
| Flavobacterium | NS | NS | 0.10 | <0.01 | 0.18 | <0.01 |
| Flavonifractor | -0.09 | <0.01 | 0.11 | <0.01 | 0.18 | <0.01 |
| Flintibacter | NS | NS | 0.05 | 0.01 | NS | NS |
| Fructilactobacillus | NS | NS | 0.04 | 0.03 | NS | NS |
| Fudania | NS | NS | NS | NS | -0.16 | <0.01 |
| Gallicola | NS | NS | -0.45 | <0.01 | -0.29 | <0.01 |
| Geobacillus | NS | NS | 0.06 | <0.01 | 0.13 | <0.01 |
| Gluconobacter | NS | NS | -0.11 | <0.01 | NS | NS |
| Gordonia | NS | NS | 0.04 | 0.03 | NS | NS |
| Gudongella | NS | NS | NS | NS | -0.15 | 0.01 |
| Halanaerobaculum | -0.13 | <0.01 | 0.11 | <0.01 | 0.17 | <0.01 |
| Holdemanella | -0.09 | <0.01 | 0.11 | <0.01 | 0.18 | <0.01 |
| Howardella | NS | NS | -0.14 | <0.01 | -0.09 | 0.04 |
| Hydrogenimonas | -0.09 | <0.01 | 0.11 | <0.01 | 0.18 | <0.01 |
| Hyphomicrobium | NS | NS | 0.04 | 0.01 | NS | NS |
| Intestinibaculum | NS | NS | -0.45 | <0.01 | -0.27 | <0.01 |
| Intestinimonas | -0.11 | 0.01 | 0.08 | <0.01 | 0.14 | <0.01 |
| Kineothrix | NS | NS | 0.04 | 0.03 | NS | NS |
| Klebsiella | -0.09 | <0.01 | 0.11 | <0.01 | 0.18 | <0.01 |
| Lachnospiracea | -0.09 | 0.03 | 0.15 | <0.01 | 0.17 | <0.01 |
| Lactiplantibacillus | 0.23 | <0.01 | -0.26 | <0.01 | -0.10 | <0.01 |
| Lactobacillus | NS | NS | NS | NS | 0.47 | <0.01 |
| Lactococcus | NS | NS | NS | NS | 0.39 | <0.01 |
| Latilactobacillus | NS | NS | 0.06 | <0.01 | NS | NS |
| Lentilactobacillus | 0.22 | <0.01 | -0.50 | <0.01 | NS | NS |
| Liberibacter | -0.10 | <0.01 | 0.12 | <0.01 | 0.20 | <0.01 |
| Ligilactobacillus | NS | NS | NS | NS | 0.35 | <0.01 |
| Limosilactobacillus | NS | NS | NS | NS | 0.21 | 0.02 |
| Luteimonas | -0.09 | <0.01 | 0.11 | <0.01 | 0.18 | <0.01 |
| Mannheimia | NS | NS | -0.15 | <0.01 | -0.18 | <0.01 |
| Massilia | -0.09 | <0.01 | 0.12 | <0.01 | 0.20 | <0.01 |
| Massiliimalia | NS | NS | 0.04 | 0.01 | NS | NS |
| Mediterraneibacter | -0.09 | <0.01 | 0.12 | <0.01 | 0.21 | <0.01 |
| Megamonas | -0.09 | <0.01 | 0.11 | <0.01 | 0.18 | <0.01 |
| Megasphaera | -0.63 | <0.01 | 0.59 | <0.01 | 1.16 | <0.01 |
| Melioribacter | 0.12 | 0.03 | -0.35 | <0.01 | -0.27 | <0.01 |
| Metamycoplasma | NS | NS | -0.10 | 0.04 | NS | NS |
| Metaprevotella | NS | NS | -0.18 | <0.01 | NS | NS |
| Methylobacterium | -0.10 | 0.02 | 0.09 | <0.01 | 0.17 | <0.01 |
| Microbacter | -0.23 | <0.01 | 0.27 | <0.01 | 0.57 | <0.01 |
| Microcella | -0.10 | 0.02 | 0.08 | <0.01 | 0.23 | <0.01 |
| Morococcus | -0.09 | <0.01 | 0.11 | <0.01 | 0.18 | <0.01 |
| Moryella | NS | NS | 0.16 | <0.01 | NS | NS |
| Mucinivorans | NS | NS | -0.21 | <0.01 | NS | NS |
| Mucispirillum | -0.10 | 0.01 | 0.08 | <0.01 | 0.20 | <0.01 |
| Murdochiella | -0.13 | <0.01 | 0.15 | <0.01 | 0.24 | <0.01 |
| Murdochiella | 0.11 | 0.03 | -0.23 | <0.01 | -0.06 | 0.03 |
| Muribaculum | NS | NS | -0.22 | <0.01 | NS | NS |
| Murimonas | -0.09 | <0.01 | 0.11 | <0.01 | 0.18 | <0.01 |
| Mycoplasma | -0.25 | <0.01 | 0.27 | <0.01 | 0.53 | <0.01 |
| Odoribacter | NS | NS | 0.08 | <0.01 | NS | NS |
| Odoribacter | -0.12 | <0.01 | 0.13 | <0.01 | 0.27 | <0.01 |
| Olsenella | -0.16 | <0.01 | 0.16 | <0.01 | 0.31 | <0.01 |
| Olsenella | -0.14 | 0.01 | -0.16 | <0.01 | -0.18 | <0.01 |
| Oscillibacter | -0.09 | <0.01 | 0.11 | <0.01 | 0.18 | <0.01 |
| Pantoea | -0.09 | <0.01 | 0.11 | <0.01 | 0.18 | <0.01 |
| Papillibacter | NS | NS | 0.04 | 0.01 | NS | NS |
| Paracoccus | NS | NS | 0.06 | <0.01 | 0.13 | <0.01 |
| Paraeggerthella | NS | NS | 0.04 | 0.01 | NS | NS |
| Paraprevotella | -0.13 | <0.01 | 0.16 | <0.01 | 0.36 | <0.01 |
| Parascardovia | -0.13 | 0.02 | 0.08 | <0.01 | -0.42 | <0.01 |
| Parasutterella | -0.09 | <0.01 | 0.11 | <0.01 | 0.18 | <0.01 |
| Parvimonas | -0.52 | <0.01 | 0.49 | <0.01 | 1.00 | <0.01 |
| Pasteurella | -0.21 | <0.01 | NS | NS | -0.22 | <0.01 |
| Pectinatus | -0.10 | <0.01 | 0.12 | <0.01 | 0.21 | <0.01 |
| Pediococcus | NS | NS | 0.12 | <0.01 | 0.13 | <0.01 |
| Pedobacter | -0.10 | 0.03 | 0.08 | <0.01 | 0.21 | <0.01 |
| Peptoanaerobacter | NS | NS | -0.13 | <0.01 | -0.15 | 0.02 |
| Peptoniphilus | -0.20 | <0.01 | -0.11 | <0.01 | NS | NS |
| Peptostreptococcaceae | NS | NS | -0.10 | 0.01 | NS | NS |
| Peptostreptococcus | NS | NS | -0.14 | 0.03 | NS | NS |
| Phocaeicola | NS | NS | 0.16 | <0.01 | NS | NS |
| Phocaeicola | -0.11 | <0.01 | 0.13 | <0.01 | 0.26 | <0.01 |
| Propionibacterium | -0.20 | <0.01 | 0.20 | <0.01 | 0.43 | <0.01 |
| Pseudoclostridium | NS | NS | 0.04 | 0.02 | NS | NS |
| Psychrobacter | NS | NS | 0.10 | <0.01 | 0.11 | <0.01 |
| Pyramidobacter | NS | NS | NS | NS | 0.14 | <0.01 |
| Rhizobium | -0.09 | <0.01 | 0.11 | <0.01 | 0.18 | <0.01 |
| Rhodococcus | NS | NS | -0.03 | 0.04 | NS | NS |
| Rikenella | -0.14 | 0.01 | 0.07 | <0.01 | NS | NS |
| Rodentibacter | NS | NS | NS | NS | -0.31 | <0.01 |
| Romboutsia | -0.09 | <0.01 | 0.11 | <0.01 | 0.18 | <0.01 |
| Roseburia | -0.09 | <0.01 | 0.11 | <0.01 | 0.18 | <0.01 |
| Roseomonas | NS | NS | 0.06 | <0.01 | 0.13 | <0.01 |
| Rothia | -0.65 | <0.01 | 0.60 | <0.01 | 1.30 | <0.01 |
| Ruthenibacterium | -0.10 | 0.02 | 0.08 | <0.01 | 0.24 | <0.01 |
| Saccharibacter | 0.13 | 0.01 | -0.46 | <0.01 | -0.11 | <0.01 |
| Saccharibacteria | 0.37 | <0.01 | -0.12 | <0.01 | -0.58 | <0.01 |
| Saccharofermentans | NS | NS | NS | NS | -0.19 | <0.01 |
| Schwartzia | -0.15 | <0.01 | 0.19 | <0.01 | 0.50 | <0.01 |
| Selenomonas | -0.51 | <0.01 | 0.52 | <0.01 | 1.09 | <0.01 |
| Shewanella | -0.10 | <0.01 | 0.12 | <0.01 | 0.20 | <0.01 |
| Shivajiella | -0.20 | <0.01 | -0.11 | <0.01 | -0.21 | <0.01 |
| Sneathia | 0.25 | <0.01 | NS | NS | 0.24 | <0.01 |
| Sphaerochaeta | NS | NS | -0.10 | <0.01 | -0.38 | <0.01 |
| Sphaerochaeta | -0.11 | <0.01 | 0.13 | <0.01 | 0.26 | <0.01 |
| Sphingobium | NS | NS | 0.08 | <0.01 | 0.14 | <0.01 |
| Sphingomonas | -0.11 | 0.01 | 0.09 | <0.01 | 0.17 | <0.01 |
| Sphingopyxis | -0.10 | 0.02 | 0.08 | <0.01 | 0.22 | <0.01 |
| Sporanaerobacter | NS | NS | 0.04 | 0.01 | NS | NS |
| Sporobacter | -0.09 | <0.01 | 0.11 | <0.01 | 0.20 | <0.01 |
| Staphylococcus | 0.23 | <0.01 | -0.16 | <0.01 | -0.27 | <0.01 |
| Streptobacillus | -0.13 | 0.02 | 0.27 | <0.01 | NS | NS |
| Streptococcus | NS | NS | NS | NS | 0.18 | 0.03 |
| Streptophyta | NS | NS | -0.14 | <0.01 | -0.27 | <0.01 |
| Syntrophococcus | 0.35 | <0.01 | -0.16 | <0.01 | 0.28 | <0.01 |
| Taibaiella | NS | NS | 0.04 | 0.05 | NS | NS |
| Tannerella | -0.16 | 0.02 | NS | NS | NS | NS |
| Tessaracoccus | -0.10 | <0.01 | 0.13 | <0.01 | 0.24 | <0.01 |
| Tessaracoccus | NS | NS | -0.40 | <0.01 | NS | NS |
| Thermus | -0.11 | 0.01 | 0.08 | <0.01 | 0.17 | <0.01 |
| Tianweitania | -0.16 | <0.01 | 0.12 | <0.01 | 0.28 | <0.01 |
| Tissierella | -0.10 | <0.01 | 0.12 | <0.01 | 0.19 | <0.01 |
| Turicibacter | -0.09 | <0.01 | 0.11 | <0.01 | 0.18 | <0.01 |
| Turicimonas | NS | NS | 0.04 | 0.02 | NS | NS |
| Unknown | -0.31 | <0.01 | 0.32 | <0.01 | 0.69 | <0.01 |
| Vagococcus | -0.09 | <0.01 | 0.10 | <0.01 | 0.18 | <0.01 |
| Vampirovibrio | -0.14 | <0.01 | 0.09 | <0.01 | 0.21 | <0.01 |
| Varibaculum | NS | NS | -0.34 | <0.01 | -0.17 | <0.01 |
| Variovorax | -0.09 | <0.01 | 0.11 | <0.01 | 0.21 | <0.01 |
| Veillonella | -0.81 | <0.01 | 0.74 | <0.01 | 1.52 | <0.01 |
| Veillonella | -0.15 | 0.02 | NS | NS | NS | NS |
| Weissella | 0.28 | <0.01 | 0.04 | 0.02 | -0.12 | <0.01 |
| Weissella | -0.11 | <0.01 | 0.11 | <0.01 | 0.19 | <0.01 |
| Williamwhitmania | 0.18 | <0.01 | NS | NS | -0.21 | <0.01 |
| Wolinella | -0.09 | <0.01 | 0.11 | <0.01 | 0.21 | <0.01 |
| Yersinia | NS | NS | -0.18 | <0.01 | NS | NS |
| Zea | 0.20 | <0.01 | NS | NS | 0.25 | <0.01 |
| NS, non-significant; FDR, false discovery rate  ^a^ Model adjusted for age, gender (male, female), smoking status (current, former, never), body mass index (BMI; <18.5, 18.5-24.9, ≥25), diabetes status (yes, no), and household income (under $15,000, $15,000-$35,000, above $35,000). | | | | | | |

Table S5. Associations of 3-year average PM_2.5_ exposure with significant oral taxa in the main analysis

| Microbiome taxa | 3-year average PM_2.5_ exposure | |
| --- | --- | --- |
|  | Log fold change^a^ | FDR |
| Acholeplasma | 0.24 | <0.01 |
| Actinomyces | 0.22 | <0.01 |
| Alkalibaculum | 0.11 | <0.01 |
| Alloscardovia | -0.16 | <0.01 |
| Aminipila | -0.10 | <0.01 |
| Anaerotaenia | 0.16 | <0.01 |
| Atopobium | 0.17 | <0.01 |
| Butyrivibrio | -0.12 | <0.01 |
| Cardiobacterium | 0.11 | <0.01 |
| Cloacibacterium | -0.11 | <0.01 |
| Corynebacterium | -0.12 | <0.01 |
| Falsiporphyromonas | 0.12 | <0.01 |
| Lactobacillus | 0.15 | <0.01 |
| Metaprevotella | 0.11 | <0.01 |
| Olsenella | 0.14 | <0.01 |
| Rikenella | 0.07 | 0.03 |
| Rodentibacter | 0.10 | 0.03 |
| Shivajiella | 0.07 | 0.03 |
| Sneathia | 0.13 | 0.04 |
| Streptobacillus | 0.08 | 0.04 |
| NS, non-significant; FDR, false discovery rate  ^a^ Model adjusted for age, gender (male, female), smoking status (current, former, never), body mass index (BMI; <18.5, 18.5-24.9, ≥25), diabetes status (yes, no), and household income (under $15,000, $15,000-$35,000, above $35,000). | | |
